# The p38*α* mitogen-activated protein kinase is a key regulator of myelination and remyelination in the CNS

**DOI:** 10.1038/cddis.2015.119

**Published:** 2015-05-07

**Authors:** S-H Chung, S Biswas, V Selvaraj, X-B Liu, J Sohn, P Jiang, C Chen, F Chmilewsky, H Marzban, M Horiuchi, D E Pleasure, W Deng

**Affiliations:** 1 Department of Biochemistry and Molecular Medicine, School of Medicine, University of California at Davis, Sacramento, CA 95817, USA; 2 Department of Oral Biology, College of Dentistry, University of Illinois at Chicago, Chicago, IL 60612, USA; 3 Department of Animal Science, College of Agriculture and Life Sciences, Cornell University, Ithaca, NY 14853, USA; 4 Institute for Pediatric Regenerative Medicine, Shriners Hospitals for Children, Sacramento, CA 95817, USA; 5 Department of Human Anatomy and Cell Science, University of Manitoba, Winnipeg, MB R3E 0J9, Canada; 6 Medical College, Hubei University of Arts and Science, Xiangyang, Hubei 441053, China

## Abstract

The p38*α* mitogen-activated protein kinase (MAPK) is one of the serine/threonine kinases regulating a variety of biological processes, including cell-type specification, differentiation and migration. Previous *in vitro* studies using pharmacological inhibitors suggested that p38 MAPK is essential for oligodendrocyte (OL) differentiation and myelination. To investigate the specific roles of p38*α* MAPK in OL development and myelination *in vivo*, we generated p38*α* conditional knockout (CKO) mice under the PLP and nerve/glial antigen 2 (NG2) gene promoters, as these genes are specifically expressed in OL progenitor cells (OPCs). Our data revealed that myelin synthesis was completely inhibited in OLs differentiated from primary OPC cultures derived from the NG2 Cre-p38*α* CKO mouse brains. Although an *in vivo* myelination defect was not obvious after gross examination of these mice, electron microscopic analysis showed that the ultrastructure of myelin bundles was severely impaired. Moreover, the onset of myelination in the corpus callosum was delayed in the knockout mice compared with p38*α* fl/fl control mice. A delay in OL differentiation in the central nervous system was observed with concomitant downregulation in the expression of OPC- and OL-specific genes such as Olig1 and Zfp488 during early postnatal development. OPC proliferation was not affected during this time. These data indicate that p38*α* is a positive regulator of OL differentiation and myelination. Unexpectedly, we observed an opposite effect of p38*α* on remyelination in the cuprizone-induced demyelination model. The p38*α* CKO mice exhibited better remyelination capability compared with p38*α* fl/fl mice following demyelination. The opposing roles of p38*α* in myelination and remyelination could be due to a strong anti-inflammatory effect of p38*α* or a dual reciprocal regulatory action of p38*α* on myelin formation during development and on remyelination after demyelination.

The myelin sheath is the fatty insulating layer that wraps around the axons of the nerves and is critical to the efficient conduction of nerve impulses. It is produced by a specialized glial cell called oligodendrocyte (OL) in the central nervous system (CNS). The proper development of OL and myelination is essential for maintaining the efficiency and speed of electrical nerve impulse. The damage to the developing OL and myelin is a hallmark of many demyelinating and dysmyelinating disorders, including the autoimmune disorders such as multiple sclerosis (MS) as well as periventricular leukomalacia, which is the predominate form of white matter injury seen in premature infants, leading to disability and neurological and cognitive impairments.^1, 2, 3^

Myelination is a complicated process involving generation of OL progenitor cells (OPCs), differentiation of OPCs into myelinating OLs, ensheathment of axons by OLs and finally wrapping the nerves with the expansion of myelin sheath.^4, 5, 6^ The study of intracellular signals that regulate myelinogenesis is crucial to our understanding of the developmental and pathological processes in white matter structures.

The mitogen-activated protein kinases (MAPKs) belong to the family of serine/threonine protein kinases that allow cells to respond to stimuli received from their extracellular environment, including mitogens as well as to intracellular stress. The p38 MAPK family members (p38*α*, p38*β*, p38*γ* and p38*δ*) in particular are implicated in various biological processes, such as cell survival, proliferation and differentiation.^7, 8, 9, 10^ The p38*α* is well established as a mediator of stress responses in neural cells; however, its physiological role(s) during OL development and myelination has only been recognized recently.^11, 12, 13, 14, 15, 16^ Using p38 inhibitors, several studies have demonstrated that p38*α* MAPK is important for myelination in cultured Schwann cells^11^ and OPCs.^12^ In addition, p38*α* has been reported to affect both cell proliferation and glial lineage progression in the presence of growth factors.^17^ More recently, Hossain *et al.*^15^ demonstrated that p38*α* controls Krox-20 to regulate Schwann cell differentiation and peripheral myelination. In contrast, p38 has also been reported as a negative regulator of Schwann cell differentiation and myelination.^16^ However, most studies were carried out using *in vitro* glial cell culture systems and with p38 inhibitors that were not selective for the p38*α* isoform. The *in vivo* molecular mechanisms and signaling events by which p38*α* regulates OPC development and myelination, therefore, remain elusive.

In an effort to identify the specific role(s) of p38*α* in myelination during early postnatal development, we have bred p38*α*-floxed (p38*α* fl/fl) mice with nerve/glial antigen 2 (NG2) or proteolipid peptide (PLP)-cre mice to generate homozygous conditional NG2/Plp-specific p38*α* knockout mice for the first time. Our data showed that p38 *α* is a positive regulator of OL development and myelination during CNS development as both myelination and OL development were impaired in specific forebrain regions of the conditional knockout (CKO) mice. Surprisingly, we observed an opposite effect of p38*α* on remyelination in the cuprizone-induced demyelination model. Our findings identified novel reciprocal roles of p38*α* during OL development in the early postnatal brain and during remyelination in adult mice, implicating the therapeutic potential of p38*α* inhibition in CNS remyelination.

## Results

### Generation of OPC-specific p38*α* knockout mice

**Figure 1:**
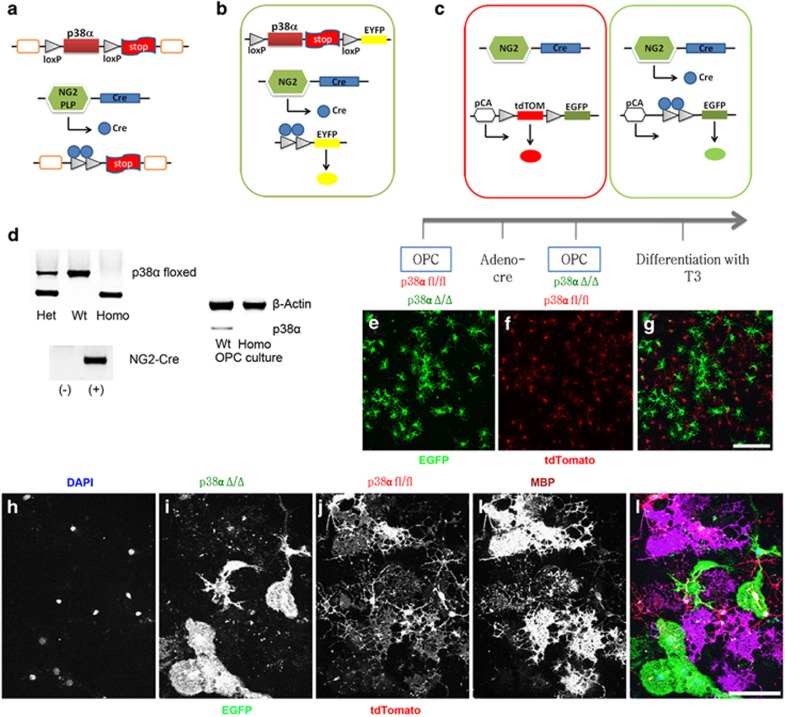
Generation of NG2/Plp^Cre^p38*α* CKO mice and *in vitro* differentiation of p38*α* CKO OPCs. (**a**) Generation of p38*α* CKO mice by crossing NG2/Plp-Cre mice and mice with p38*α*-floxed alleles (p38*α* fl/fl). Schematic diagram showing the targeted genetic locus of p38*α* gene flanked by two loxP sites. p38*α* gene deletion is driven by Cre-loxP recombination that is under the control of NG2/Plp promoter activity in targeted cells. (**b** and **c**) Schematic illustrations demonstrating the EYFP (**b**) and ROSA26tdTomato (**c**) reporter system. Under the control of NG2/Plp promoter, Cre recombination results in an excision of tdTomato (mT) and the expression of EGFP (mG) in NG2/Plp-positive cells. (**d**) Genotyping of NG2-Cre mice demonstrating homozygosity of p38*α*-floxed allele and positive expression of NG2-Cre transgene. Western blotting analysis confirmed that the p38a protein is absent in p38*α* CKO OPC cultures. (**e**–**g**) Schematic diagram of *in vitro* differentiation of purified primary OPCs from CKO mice. Four days after culture, both p38*α* CKO (green: **e**) and p38*α* fl/fl cells (red: **f**) were observed. (**h**–**l**) Immunohistochemistry for anti-MBP after terminal differentiation of OPCs into myelin-forming OLs. MBP immunofluorescence labeling demonstrates that MBP expression was diminished in p38*α* CKO cells (green cells in panels (**i**) and (**l**)), while p38*α* fl/fl cells express MBP (red cells in panels (**j**) and (**l**)). Scale bars: panel (**g**)=500 *μ*m; panel (**l**)=50 *μ*m

To identify the specific *in vivo* role(s) of p38*α* in OL development and myelination, we have generated two conditional OPC-specific p38*α* knockout mice by crossing NG2-Cre or plp-Cre mice with p38*α*-floxed (p38*α* fl/fl) mice.^18^ The targeted disruption of the p38*α* gene in NG2^cre^/p38*α*^−/−^ or Plp^cre^p38*α*^−/−^ (p38*α* CKO) mice was mediated by Cre-loxP recombination under the control of the NG2 or the Plp promoter. The p38*α* gene deletion only occurred in specific cell types with an active NG2 or Plp promoter as illustrated (Figure 1a). We also utilized a reporter strain (EYFP and ROSA26-mT/mG) to simultaneously validate Cre recombination in these cells (Figures 1b and c). The genotype of NG2-Cre p38*α* CKO mice was confirmed by the presence of homozygous p38a-floxed alleles and being positive for NG2-Cre transgene (Figure 1d). The p38*α* CKO mice were viable with no obvious abnormalities at the gross phenotypic level.

### *In vitro* myelination is blocked in the NG2 p38*α* CKO-derived OLs

We first examined whether endogenous p38*α* deletion in OPCs affected their ability to form myelin after differentiation *in vitro*. To achieve this, we first generated OPC cultures using pups from cross breed of homozygous ROSA26-tdTomato-EGFP (enhanced green fluorescent protein) and NG2-Cre mice, in which, tdTomato gene is excised by Cre recombinase, leading to EGFP expression in cells with a constitutive NG2 promoter (Figures 1e and g). After OL cultures were made from p38afl/fl and NG2-Cre p38*α* mice, we confirmed for the absence of p38a protein expression in cultures (Figure 1d). Purified OPCs from p38*α* CKO and p38*α* fl/fl mice were differentiated *in vitro* for 4 days (Figures 1e and g). Consistent with the previous studies,^11, 12^ the expression of myelin basic protein (MBP), a major protein of central and peripheral myelin, was completely diminished in the p38*α* CKO OLs (green cells in Figures 1i and l), whereas p38*α* fl/fl OLs expressed MBP (red cells in Figures 1j and l). This result further confirmed a critical role of p38*α* in normal myelin production at least *in vitro*.

### Myelination defects in p38*α* CKO mice

**Figure 2:**
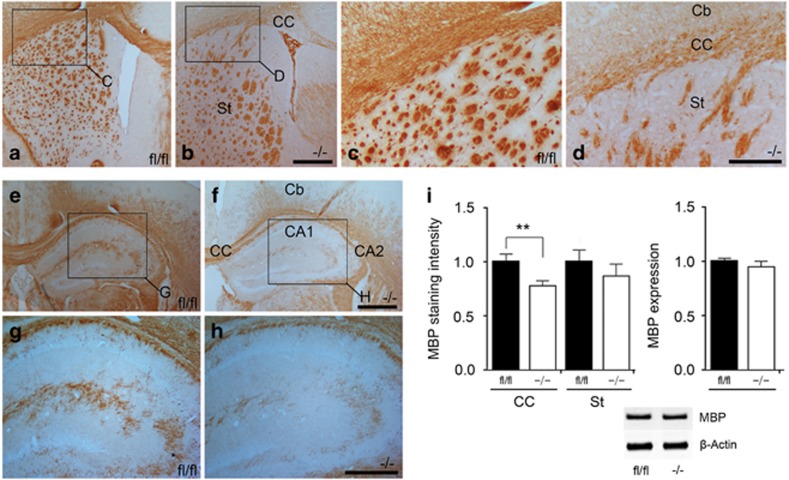
No obvious gross myelination defects were observed in p38*α* CKO mice. (**a**–**d**) Transverse sections were peroxidase stained for anti-MBP in p38*α* fl/fl (**a** and **c**) and p38*α* CKO (**b** and **d**) at P28. Panels (**c**) and (**d**) are higher magnification views of the regions indicated in panels (**a**) and (**b**). (**e**–**h**) Transverse sections peroxidase stained for anti-MBP in p38*α* fl/fl (**e** and **g**) and p38*α* CKO (**f** and **h**) at P28. Panels (**g**) and (**h**) are higher magnification views of the regions indicated in panels (**e**) and (**f**). MBP expression in some areas of the CC seemed to be relatively weak in the knockout compared with p38*α* fl/fl, but generally the difference was not obvious at low magnification of light microscope level. (**i**) Statistical analysis of the MBP staining intensity in the CC (*P*<0.01) and St of P28 p38*α* CKO and p38*α* fl/fl mice. Western blotting analysis of MBP protein in the whole brains of P28 p38*α* CKO and p38*α* fl/fl mice. **P<0.01. Abbreviation: Cb, cerebral cortex. Scale bars: panel (**b**)=500 *μ*m; panel (**d**)=250 *μ*m; panel (**f**)=500 *μ*m; panel (**h**)=250 *μ*m

We next sought to investigate the extent of *in vivo* myelination in the neonatal p38*α* CKO mice. Surprisingly, under the light microscope, dense and compact MBP staining was seen throughout the white matter regions of the p38*α* CKO brain. Although the MBP staining intensity in some areas of the corpus callosum (CC) tended to be weaker in the CKO compared with p38*α* fl/fl, the differences in general were not significant at low magnification (Figure 2). The mean MBP staining intensity was 78.1±5% (compared with same regions in p38*α* fl/fl assigned as 100%, analyzed from 117 different observation fields, *N*=12, *P*=0.008) in the CC and 87.5±11% (analyzed from 83 different observation fields, *N*=7, *P*=0.449) in the striatum (St) compared with the p38*α* fl/fl mouse brains. Western blotting analysis of the whole brain between the p38*α* CKO and p38*α* fl/fl did not show any noticeable difference in the MBP expression (Figure 2i).

**Figure 3:**
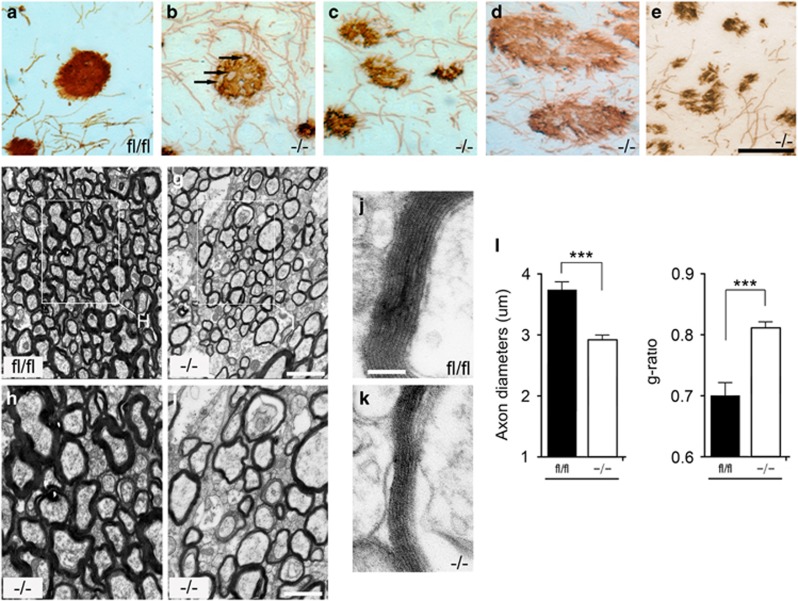
p38*α* CKO mice show morphological defects and reduced axon diameters. (**a**–**e**) Sagittal sections were peroxidase stained for anti-MBP in p38*α* fl/fl (**a**) and p38*α* CKO (**b**–**d**) at P12. Axonopathies observed in the p38*α* CKO: axonal swelling (**b** and **c**), changes in the density of axons (**c** and **d**) and degeneration (**e**) were frequently seen. (**f**–**k**) Electron microscopic analysis of myelinated bundles in CC sections from P12 p38*α* CKO and p38*α* fl/fl mice. Panels (**h**) and (**i**) are higher magnification views of the regions indicated in panels (**f)** and (**g**). The thickness of myelin bundle diameter surrounding the axons was significantly reduced in the p38*α* CKO (**k**) compared with p38*α* fl/fl (**j**). (**h**) and (**i**) are higher magnification views of the regions indicated in panels (**f**) and (**g**). (**l**) Quantification of axon diameters and *g*-ratio (the ratio of axon diameter to fiber diameter). The axon diameters were significantly reduced in the CKO CC (*P*<0.0001). The *g*-ratio was significantly increased from 0.693 in p38*α* fl/fl to 0.818 in p38*α* CKO mice (*P*<0.0001). ***P<0.005. Scale bars: panel (**e**)=50 *μ*m; panel (**g**)=5 *μ*m; panel (**h**)=2.5 *μ*m; panel (**j**)=1 *μ*m

However, a detailed morphological observation using electron microscopy revealed several characteristic myelination and axonal defects in the p38*α* CKO brain. We observed axonal swellings (Figure 3b: arrows), changes in the axon density (Figures 3c and d) and axon degeneration (Figure 3e) at postnatal day (P) 12. These morphological changes that were indicative of degenerating axons suggest that p38*α* has an important role in establishing complete and functional axons. In p38*α* CKO, the thickness of the myelin sheath surrounding the axons was significantly reduced compared with p38*α* fl/fl mice (Figures 3j and l). Myelin thickness was measured as the g-ratio (the ratio of axon diameter to fiber diameter). A total of 380 axons from 7 mice were investigated from different non-overlapping observational fields (OF). The average thickness of axons from p38*α* CKO mice was 2.91±0.07 *μ*m (348 OF, *N*=8, *P*<0.0001 compared with p38*α* fl/fl), while axons from p38*α* fl/fl mice were typically 3.73±0.14 *μ*m thick (126 OF, *N*=6). The average *g*-ratio of the nerve fibers from p38*α* CKO mice was 0.818±0.01 (276 OF, *N*=8) compared with 0.693±0.02 (234 OF, *N*=6) in p38*α* fl/fl mice, thus suggesting a significant thinner myelin sheath in p38*α* CKO (*P*<0.0001 compared with p38*α* fl/fl). The myelination defect phenotype is mainly observed in early stage of myelination, and the phenotype is less obvious in adult/old p38*α* CKO mice. The average thickness of axons from P90 p38*α* CKO mice was 4.59±0.63 *μ*m (87 OF, *N*=3, *P*<0.0001 compared with p38*α* fl/fl), while axons from p38*α* fl/fl mice were typically 5.27±0.54 *μ*m thick (43 OF, *N*=2).

### P38*α* CKO results in a delay in the onset of myelination in the CC

**Figure 4:**
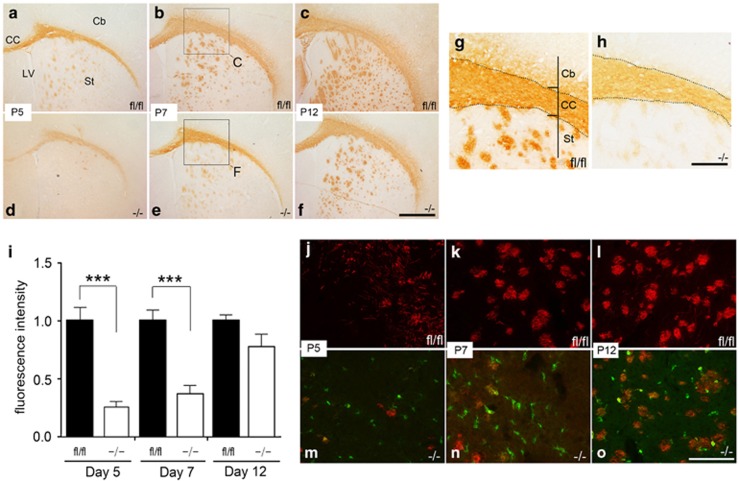
Delay in the onset of myelination in the CC of p38*α* CKO mice. (**a**–**h**) Transverse sections peroxidase stained for anti-MBP at P5, P7 and P12 in p38*α* fl/fl and p38*α* CKO revealed that the onset and progression of myelination were delayed in the p38*α* CKO. Panels (**g**) and (**h**) are higher magnification views of the regions indicated in panels (**b**) and (**e**). (**i**) Quantitative analysis of MBP expression during different postnatal periods in the CC of the p38*α* fl/fl and p38*α* CKO mice, suggesting a delay in onset of myelination (P5 and P7 *P*<0.0001). (**j**–**o**) Immunofluorescence-stained sections with anti-EYFP (green) and MBP (red) showed that the onset and progression of myelination were delayed in the p38*α* CKO CC. Green cells represent p38*α* knockout cells. Abbreviations: Cb, cerebral cortex; LV, lateral ventricle. ***P<0.005. Scale bars: panel (**f**)=250 *μ*m; panel (**h**)=75 *μ*m; panel (**o**)=250 *μ*m

As we observed that the ultrastructural myelination defects in the p38*α* CKO were more pronounced in early stages of myelination, we studied the myelination pattern at several early embryonic developmental stages. To identify a myelination phenotype during development in the p38*α* CKO, we directly compared the intensity and pattern of MBP staining in brain sections from P5, P7 and P12 from p38*α* CKO and p38*α* fl/fl control mice. In P5 p38*α* fl/fl brains, strong MBP immunostaining was seen in the CC, as expected (Figure 4a). In contrast, the intensity of MBP immunostaining in the CC was significantly weaker in the p38*α* CKO (Figure 4d). In the p38*α* CKO St, onset of myelination was observed (Figure 4a), while onset of myelination had not taken place in most areas of the St in p38*α* fl/fl (Figure 4d). At P7, the MBP-positive band in the CC was found to be thinner in p38*α* CKO mice (Figures 4e and h) compared with p38*α* fl/fl control (Figures 4b and g). In addition, the intensity of MBP staining in the CC was significantly less in the p38*α* CKO CC (Figures 4e and h) compared with p38*α* fl/fl (Figures 4b and g). However, at P12, the intensity of MBP staining in the CC between the p38*α* fl/fl and p38*α* CKO brain became comparable (Figures 4c and f). The fluorescence intensity of MBP staining in the CC was quantified in numerous non-overlapping OF (Figure 4i). The fluorescence intensity of MBP staining at P5 was 24.9±6.2% (237 OF, *N*=13, *P*<0.0001), at P7 37.2±7.5% (177 OF, *N*=9, *P<*0.0001) and at P12 82.9±13% (274 OF, *N*=17, *P=*0.23), compared with p38*α* fl/fl control (521 OF, *N*=34, assigned as 100%). Next we investigated whether these myelination defects are the direct result of knocking out p38*α* from cells. The EYFP reporter system allows us to visualize the cells when the p38*α* gene is excised out and Cre is expressed in the cells.

**Figure 5:**
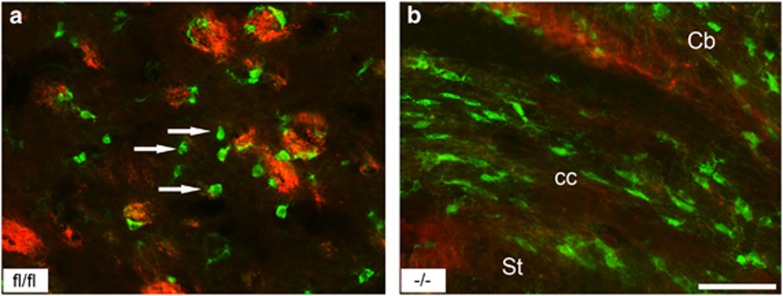
The p38*α* knockout cells show myelination defects *in vivo*. Immunofluorescence-stained sections with anti-EYFP (green) and MBP (red) in the (**a**) St and (**b**) CC of p38*α* CKO mice. Green cells represent p38*α* knockout cells. Majority of p38*α* knockout cells (arrows) did not express MBP. Abbreviation: Cb, cerebral cortex. Scale bar: panel (**b**)=100 *μ*m

Immunostained sections of p38*α* CKO CC using anti-EYFP (green) and anti-MBP antibodies (red) showed that the onset and progression of myelination are delayed at different developmental stages (Figures 4j and o). The green cells (p38*α* knockout cells) generally did not overlap with the MBP staining (red), suggesting that knockout cells produced none or negligible myelin compared with p38*α* fl/fl cells at similar developmental stages of myelination (Figures 4j, o and 5).

### OPC differentiation is delayed in p38*α* CKO

**Figure 6:**
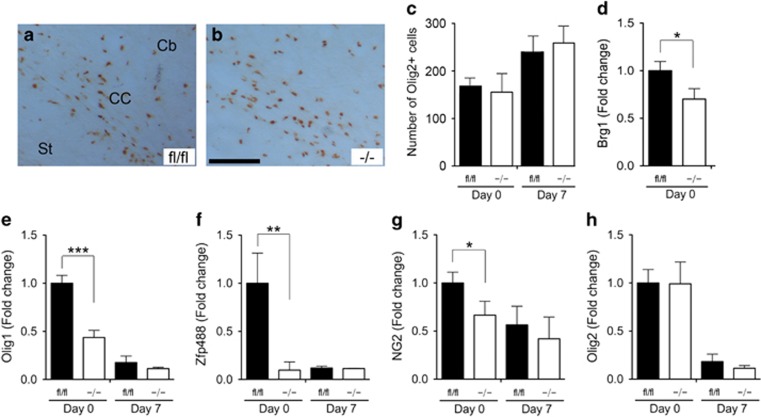
p38*α* gene deletion results in a delayed OL differentiation without affecting proliferation. (**a** and **b**) Transverse sections were peroxidase stained for anti-Olig2 at P7 in the CC of the p38*α* fl/fl (**a**) and p38*α* CKO mice (**b**). (**c**) Quantification of Olig2-immunopositive cells in the CC at P0 and P3 in the p38*α* fl/fl and p38*α* CKO. The numbers of Olig2-posivtive cells were unchanged. (**d**–**h**) Quantitative PCR analysis for Brg1 (**d**), Olig1 (**e**), Zfp488 (**f**), NG2 (**g**) and olig2 (**h**) at P0 and P7 in the p38*α* fl/fl and p38*α* CKO mice. Olig1 and Zfp488 expression is significantly downregulated in the P0 p38*α* CKO mice (**e** and **f**). *P<0.05, **P<0.01 ***P<0.005. Abbreviation: Cb, cerebral cortex. Scale bar: panel (**b**)=100 *μ*m

We then assessed whether the deletion of p38*α* gene affects the proliferation of OPCs that in turn would lead to myelination defects in the p38*α* CKO mice. The numbers of Olig2-positive cells in the CC of p38*α* fl/fl and p38*α* CKO mice were examined at P0 and P7. Olig2-positive OPC numbers were unchanged in the p38*α* CKO at both time points (Figures 6a and c). Quantitatively, at P0, the numbers of Olig2-positive cells were 168±14 in p38*α* fl/fl (223 areas were examined, *N*=8) *versus* 154±47 in p38*α* CKO (276 areas were examined, *N*=12, *P*=0.81); and at P7 the numbers of Olig2-positive cells were 237±12 in p38*α* fl/fl (186 areas were examined, *N*=8) *versus* 257±13 in p38*α* CKO (263 areas were examined, *N*=11, *P*=0.31). These results suggested that p38*α* did not have a role in OPC proliferation during perinatal and postnatal development.

We next examined the effects of p38*α* deletion on the expression of various stage specific OPC and OL mRNAs, including Brg1, Olig1, Olig2, Zfp488 and NG2, using qPCR analysis. It has been recently shown that Smarca4/Brg1 is necessary and sufficient to initiate and promote OL lineage progression and maturation.^16^ Basic helix-loop-helix transcription factors Olig1 and Olig2 have been shown to regulate OL development.^19, 20^ Zfp488 is an OL-specific zinc finger protein, identified as a downstream effector of Olig1, which physically cooperates with Olig2 during OL differentiation.^21, 22, 23^ NG2 is a chondroitin sulfate proteoglycan and NG2+ cells have been recognized as OPCs. Our qPCR expression results showed at P0 there was significantly reduced expression of Brg1 (100±11% in p38*α* fl/fl *versus* 71.2±14% in p38*α* CKO, *P*=0.039), Olig1 (100±7.6% in p38*α* fl/fl *versus* 44.3±6.2% in p38*α* CKO, *P*<0.0001), Zfp488 (100±32% in p38*α* fl/fl *versus* 8.21±7.8% in p38*α* CKO, *P*=0.013) and NG2 (100±11% in p38*α* fl/fl *versus* 65.2±13% in p38*α* CKO, *P*=0.048) mRNAs (Figures 6d and h). However, downregulation of Olig1, Zfp488 and NG2 expression in the p38*α* CKO was restricted to an early stage of OPC differentiation (P0). By P7, the expression levels of the following genes were comparable between the p38*α* fl/fl and CKO mice: Olig1, 15.6±6.1% in p38*α* fl/fl *versus* 10.2±0.6% in p38*α* CKO, *P*=0.391; Zfp488 11.5±0.69% in p38*α* fl/fl *versus* 10.3±0.2% in p38*α* CKO, *P*=0.113; and NG2, 56.2±18% in p38*α* fl/fl *versus* 42.6±20% in p38*α* CKO, *P*=0.625. Olig2 expression was not significantly different between the p38*α* CKO and p38*α* fl/fl both at P0 and P7 (Figure 6h). These results suggest that p38*α* gene deletion affected OPC differentiation without affecting proliferation, and this, in turn, might contribute to a delayed onset of the myelination.

### p38*α* CKO promotes remyelination after cuprizone-induced demyelination

**Figure 7:**
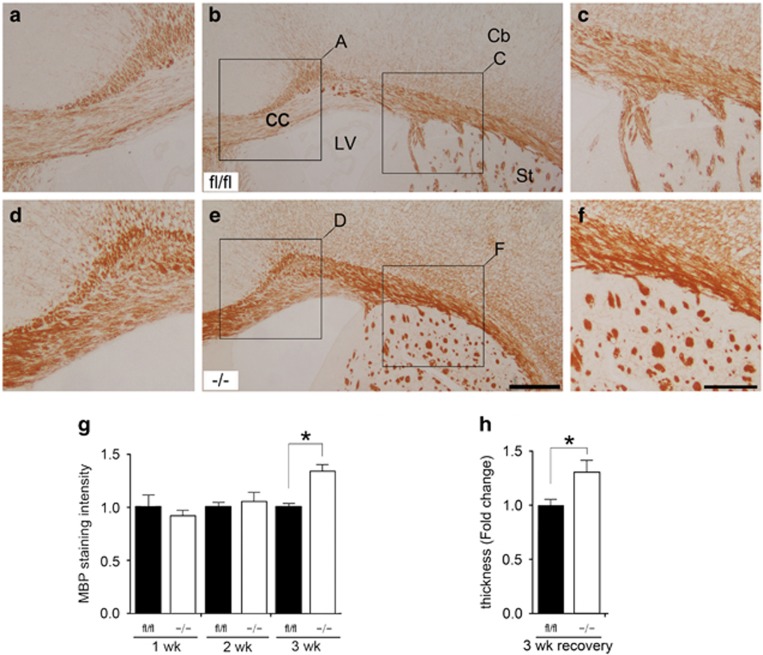
p38*α* CKO promotes remyelination after cuprizone-induced demyelination. (**a**–**f**) Transverse sections peroxidase stained with anti-MBP showing remyelination process in the Cc after 3 weeks following discontinuation of cuprizone intake in the p38*α* fl/fl (**a**–**c**) and p38*α* CKO (**d**–**f**) mice. Panels (**a** and **c**) and (**d** and **f**) are higher magnification views of the regions indicated in panels (**b**) and (**e**), respectively. MBP expression is significantly increased in the CC of p38*α* CKO (**d**–**f**) compared with p38*α* fl/fl (**a**–**c**) mice. (**g** and **h**) Quantification of MBP staining intensity (**g**) and thickness (**h**) during a recovery period in the p38*α* fl/fl and p38*α* CKO. p38*α* CKO mice showed a better remyelination ability compared with p38*α* fl/fl during the remyelination process. *P<0.05. Abbreviations: Cb, cerebral cortex; LV, lateral ventricle. Scale bar: panel (**e**)=500 *μ*m; panel (**f**)=250 *μ*m

We next examined the specific role of p38*α* isoform in the cuprizone-induced demyelination/remyelination model. Mice that were on cuprizone diet for 5 weeks progressively lost myelin as indicated by the loss of MBP immunostaining in the CC. When mice were returned to normal diet, immediate remyelination and recovery process began. To identify the remyelination phenotype, a peroxidase immunohistochemistry with anti-MBP antibody was performed. Unexpectedly, we observed a reverse effect of p38*α* on remyelination in cuprizone-induced demyelination model. The p38*α* CKO mice showed better remyelination ability than the p38*α* fl/fl mice (Figure 7). The intensity of MBP staining was similar after 1 week of recovery between the p38*α* fl/fl (100±12%) (223 different areas were examined, *N*=16) and p38*α* CKO brains (91±8.0%) (178 different areas were examined, *N*=13, *P*=0.565) and also, after 2 weeks, 100±7.3% in p38*α* fl/fl (198 different areas were examined, *N*=14) *versus* 106±6.1% in p38*α* CKO (246 different areas were examined, *N*=17, *P*=0.530). However the remyelination ability was significantly increased in p38*α* CKO mice after the third week of recovery. MBP staining intensity in p38*α* fl/fl was 100±4.4% (179 different areas were examined, *N*=12) *versus* 134±13% in p38*α* CKO (231 different areas were examined, *N*=14, *P*=0.030). In addition, the thickness of MBP-stained axon bundles in the p38*α* CKO CC was increased 1.32-fold (132±12%) (215 different areas were examined 215, *N*=16) compared with the p38*α* fl/fl control (100±6.2%, 193 different areas were examined 193, *N*=15, *P*=0.031).

## Discussion

In this study, we report the specific *in vivo* role of the p38*α* MAPK isoform in myelin formation during development, as well as during remyelination, for the first time, by generating OL-specific p38*α* CKO mice. Our main finding was that, although a myelination phenotype was not evident at a gross level, there were several myelination defects at the ultra-structural level. Specifically, myelin bundles in the CC failed to develop normally, and there was a delayed onset of myelination in the CC. These defects could be partly due to a delay in OL differentiation during postnatal development as OPC proliferation remained normal in these knockout mice. This was supported by our observation that gene expression levels of several critical transcription factors of OPC maturation such as Olig1, Zfp488, and the OPC marker NG2 were significantly downregulated during early neonatal development in these knockout mice. Additionally, similar to previous reports, an inherent myelination defect was apparent in the primary OPCs isolated from p38*α* CKO mouse brains. These OPCs failed to synthesize MBP when differentiated *in vitro*. Our second major finding was that p38*α* appears to have a negative regulatory role during remyelination in cuprizone-induced demyelination model in adult p38*α* CKO mice. The p38*α* CKO mice showed enhanced remyelination ability compared with p38*α* fl/fl during the recovery period.

Understanding the intracellular signals that regulate myelination during development or following demyelination is crucial for identifying developmental and pathological processes in several dysmyelinating disorders, including leukodystrophies, and demyelinating disorders, such as MS. The p38 MAPK signaling pathway has been initially shown to be activated by stress stimuli and to induce inflammatory responses.^7, 8, 9, 10^ Recent studies have recognized its critical role in myelination but mostly in the peripheral nervous system. Initially, Fragoso *et al.*^11^ showed that p38 inhibition by p38 MAPK pharmacological agents blocks myelin formation in cultured Schwann cells. Forskolin-induced MBP gene expression was also blocked by the p38 inhibitor SB203580.^13^ Hossain *et al.*^15^ demonstrated that p38*α* MAPK controls Krox-20 to govern Schwann cell differentiation and peripheral myelination. In addition, inhibition of p38 with PD169316 and SB203580 blocked accumulation of protein and mRNA of several cell-stage-specific markers characteristic of differentiated OLs, including MBP.^12^ Recently, Cui *et al.*^24^ showed that sphingosine 1-phosphate receptor modulates human OL differentiation by activating ERK1/2 and p38 MAPK signaling. These previous studies support p38 MAPK as a positive regulator of OL differentiation and myelination. However, SB203580, the most widely used p38 MAPK inhibitor, is not p38*α* specific as it blocks both p38*α* and p38*β* isoforms and has been reported to target additional proteins, albeit at higher concentrations.^25, 26^ There is a report showing that p38*α* and p38*γ* appear to have opposite functions in skeletal muscle differentiation. p38*α* has been shown to be a strong promoter of skeletal muscle differentiation, whereas p38*γ* may act as a negative regulator.^27^ Thus the use of nonspecific inhibitors has limitations in revealing a specific role of individual p38 MAPK isoforms. To our knowledge, our study is the first report aimed at identifying a specific *in vivo* role of p38*α* in OL development and myelination in the CNS.

Our observations with the primary OPC from p38*α* CKO mice is consistent with the previous studies which demonstrated that myelination was completely inhibited in primary OPC culture from the p38*α* CKO mice.^12^ However, the gross *in vivo* p38*α* CKO myelin phenotype was not very discernable compared with p38*α* fl/fl mouse brains. This discrepancy might be due to the redundant roles of other isoforms of p38 MAPK (p38*β*, p38*γ* and p38*δ*). For example, the two isoforms, p38*α* and p38*β* are ~70% identical in their amino-acid sequence, show similar substrate specificity and demonstrate overlapping functions that are critical during mouse embryonic development.

The underlying mechanism of how p38*α* controls OL differentiation and myelination has not been fully elucidated. Weider *et al.*^28^ initially identified an ATP-dependent SWI/SNF chromatin-remodeling enzyme Smarca4/Brg1 to be required for Schwann cell differentiation and myelination. Recently, Yang *et al.*^16^ identified that Smarca4/Brg1 is necessary and sufficient to initiate and promote OL lineage progression and maturation. They identified Olig2 as a prepatterning factor that directs the recruitment of Brg1 to OL lineage-specific *cis*-regulatory elements during the critical transition from OPCs to OLs. Our analysis shows that Brg1 is significantly reduced in the p38*α* CKO cells at P0. Interestingly, in myogenic cell differentiation, p38MAPK appears to regulate Brg1 expression and governs myogenin expression, which has the ability to convert some of the non-muscle cells into the myogenic lineage. Supporting this idea, Li *et al.*^29^ found that the p38 MAPK is required for BRG1 recruitment in 12-*O*-tetradecanoylphorbol-13-acetate-mediated myogenin induction. Thus we speculate that p38*α* directly regulates Brg1 at early stages of OPC differentiation and myelination. Further studies are warranted to identify the underlying mechanisms by which p38*α* controls Brg1 in OL differentiation.

An unexpected role of p38*α* in remyelination was observed in cuprizone-induced demyelination in adult mice. In this model, p38*α* deletion in OPCs caused hypermyelination following demyelination, as evident by increased thickness of MBP-stained axon bundles compared with p38*α* fl/fl control. This suggests that p38*α* may have a tonic negative regulatory role controlling myelin arrest in adult mice. Overall, our study suggests that p38*α* could have a dual role as regulator of myelination: during development it acts as a pro-differentiation factor, and but in the adult's brain it participates in myelin arrest. Another possible cause for increased myelination in p38*α* mice could be due to the anti-inflammatory effect resulting from deletion of p38*α*. Consequently, p38*α* could be an interesting pharmaceutical target because of its critical role in inflammatory diseases, such as psoriasis and arthritis. There is an extensive evidence that the p38 MAPK signaling pathway contributes to the proinflammatory cytokine overproduction in many disease states. Cytokine overproduction may contribute towards many neurodegenerative disorders.^30^ Supporting this, there is a report that a p38*α*-specific inhibitor, UR-5269, may have therapeutic role in a widely used mouse model of the MS, experimental autoimmune encephalomyelitis.^31^ Several human clinical trials for diseases such as rheumatoid arthritis, asthma, atherosclerosis and acute lung injury are underway.^32^

Taken together, our studies reveal a specific *in vivo* role of p38*α* in OL development and myelination in the CNS. Opposite roles of p38*α* raise a therapeutic possibility of the p38*α* CKO inhibition in myelin-deficiency diseases, such as MS and periventricular leukomalacia, but further studies are necessary to further define the underlying mechanisms. Nonetheless, the present study clearly identifies p38*α* as a key regulator of myelination and remyelination in the CNS.

## Materials and Methods

### Generation and genotyping of transgenic p38*α*^fl/fl^ and NG2/Plp^cre^ p38*α*^−/−^ (p38*α* CKO) mice

Generation of mice with floxed alleles of p38*α* (B6.129-Mapk14<tm1.2Otsu>) has been described previously.^18^ NG2/Plp-cre recombinase-expressing mice as well as the recombination-reporter strain ROSA26-tdTomato (mT)-EGFP (mG) (Gt (ROSA) 26-Sortm4 (ACTB-tdTomato,-EGFP)Luo/J) were obtained from the Jackson Laboratory (Bar Harbor, ME, USA). Conditional NG2/Plp-specific p38*α* mice were generated using Cre/loxP recombination system by cross breeding NG2/Plp-Cre mice (FVB/N background) and p38*α*-floxed (p38*α* fl/fl) mice (C57BL/6 background). The NG2/Plp-Cre-positive heterozygous p38*α* mice were then backcrossed to p38*α* fl/fl mice to obtain mice homozygous for the p38*α* fl/fl with and without NG2/Plp-Cre. Mice were genotyped using tail-tip DNA by using the QIAamp DNA Mini Kit (Qiagen, Valencia, CA, USA). The genotypes of p38*α* fl/fl and p38*α* CKO mice were examined by analyzing two transgenic compositions in genomic DNA, including NG2/Plp-Cre and the presence of p38*α*-floxed alleles, using specific primers. All mice were maintained in accordance to the NIH guidelines for the Care and Use of Laboratory Animals. Experimental protocols used for this study were approved by the Institutional Animal Care and Use Committee at the University of California, Davis. The Cre-LoxP recombination rate of NG2 Cre p38*α* CKO was ~57% and that of Plp Cre p38*α* CKO was ~63%.

### Mixed glial cultures

Primary cultures of mixed glial cells were established from p38*α* CKO and p38*α* fl/fl (control) as previously reported.^33^ In brief, the brains from P0 mice were removed and submerged in ice-cold Leibovitz L-15 medium. The Olfactory bulbs, cerebral cortex, and hindbrains were removed. Meninges with blood vessels and choroid plexus were carefully peeled off. Remaining tissues were cut into small pieces and digested using trypsin (0.0625% w/v) in HBSS for 20 min. Cells were dissociated by trituration and collected by centrifugation at 400 × *g* for 5 min. Dissociated cells were suspended in MEM alpha containing FBS (10% v/v) and plated in a tissue culture dish. Cells were maintained in growth medium (GM), a mixture of N1 medium (high glucose DMEM supplemented with 6 mM l-glutamine, 10 ng/ml biotin, 5 *μ*g/ml insulin, 50 *μ*g/ml apo-transferrin, 30 nM sodium selenite, 20 nM progesterone and 100 *μ*M putrescine) and B104 neuroblastoma-conditioned medium (7 : 3 mixture v/v). Media was changed daily, and the OPCs cultures were grown to confluency before purification.

### Immunopanning for purification of primary OPCs

Purification of OPCs was performed using a two-step approach of negative and positive affinity selection using surface markers. Separate low-adhesion dishes (10 cm) were first coated overnight with secondary antibodies that bind rat IgG or mouse IgG. These plates with secondary antibodies were then incubated with the respective primary antibodies: either anti-Thy1 rat IgG (clone 30H12) or anti-NG2 mouse IgG at least 2 h before washing to remove unattached immunoglobulins. Mixed glial cultures were then trypsinized and resuspended in N1 medium containing 0.1% IgG-free BSA and plated on the anti-Thy1 IgG dish and incubated in a 37 °C, 5% CO_2_ humidified environment for a 30-min negative selection. The non-adherent cell population from this dish was then removed and plated on the anti-NG2 IgG dish and incubated in a 37 °C, 5% CO_2_ humidified environment for a 30-min positive selection. Adherent cells that represent primary OPCs were then collected by a rapid trypsinization using 0.5% Trypsin solution followed by buffered, purified soybean trypsin inhibitor (Gibco, Life Technologies, CA, USA, 500 *μ*g/ml in HBSS). OPCs were then plated in poly-L lysine-coated plates with N1 medium supplemented with FGF2 (10 ng/ml) and PDGF-A (5 ng/ml).

### Differentiation of OPCs to OLs

To induce *in vitro* differentiation of OPCs, the culture medium was switched from GM to OL differentiation medium (a 1 : 1 mixture of high glucose DMEM and Ham's F-12 supplemented with 4.5 mM L-glutamine, 10 ng/ml biotin, 12.5 *μ*g/ml insulin, 50 *μ*g/ml transferrin, 24 nM sodium selenite, 10 nM progesterone, 67 *μ*M putrescine, 0.4 *μ*g/ml 3,5,3′5′-tetraiodothyronine, 100 units/ml penicillin and 100 *μ*g/ml streptomycin). Cultures were allowed to differentiate for 4 days, a time point at which >80% of the control OLs from p38*α* fl/fl mice were positive for MBP.

### RNA isolation and qPCR

The relative expression of transcription factors and markers associated with myelination in p38*α* CKO and p38*α* fl/fl OPCs and OLs were analyzed by qPCR. Total RNA was extracted from cultures of OPCs and OLs and was reverse-transcribed to cDNA using Multiscribe reverse transcriptase (Applied Biosystems, Foster City, CA, USA). Subsequent qPCR estimations were performed using Taqman primer-probe sets (Life Technologies) for Olig1 (Mm00497537_s1), Olig2 (Mm01210556_m1), Zfp488 (Mm02763085_s1), NG2 (Mm00507257_m1), CNPase (Mm01306640_m1), MPB (Mm01266402_m1) and PLP (Mm00456892_m1) in triplicate using a Lightcycler 480 (Roche Applied Science, Pleasanton, CA, USA). A relative efficiency plot was constructed for each gene to compare target and reference *Δ*Cp values and ensure that the absolute slope of fit line is <0.1.

### Cuprizone-induced demyelination

CNS demyelination was induced by cuprizone in mice according to an established protocol.^34^ Eight-week-old p38*α* CKO and p38*α* fl/fl male mice were fed either 0.2% cuprizone (w/w) (Lab Die, Richmond, IN, USA) or control pellet diets *ad libitum* for 5 weeks to induce demyelination. Food pellets were changed on alternate days, and body weight was recorded weekly. After 5 weeks, mice were returned to a regular diet to allow for remyelination.

### Electron microscopy

Mice were perfused, and the brains were fixed overnight in Karnovsky's fixative (5% glutaraldehyde + 4% PFA in 0.08 m phosphate buffer), followed by postfixing in 2% osmium tetroxide in 0.1 m cacodylate buffer. Samples were subsequently dehydrated, placed in propylene oxide and then embedded in epon. Semi-thin (1 *μ*m) sections were stained with toluidine blue to aid in orientation of white matter tracts. Ultrathin sections (70 nm) of the regions of interest were cut and collected on Formva-coated single slot copper grids. Sections were stained with uranyl acetate and lead citrate and examined in a Philips CM120 Electron Microscope (Hillsboro, OR, USA) at 80 kV. Low magnification images were taken to view the axon distributions, and high magnification images were obtained to show the myelin sheath layers for *g*-ratio calculation. Images were acquired via a high-resolution CCD camera (Gatan, Pleasanton, CA, USA) and processed in DigitalMicrograph (Gatan). Images were imported to Adobe Photoshop (San Jose, CA, USA) for adjusting the brightness and contrast and composing figures.

### Immunoblotting

Western blotting analysis was carried out on total protein extracts from the whole brains of p38*α* CKO mice. Briefly, electrophoresis was performed in SDS-polyacrylamide gel, using 30 *μ*g of proteins per lane. The following primary antibodies were used: mouse anti-MBP antibody (1 : 1000; Sternberger and Sternberger, Baltimore, MD, USA), rabbit polyclonal p38*α* antibody (1 : 200, Santa Cruz Biotechnology, Santa Cruz, CA, USA) and mouse anti-*β*-Actin monoclonal antibody (1 : 1000, Santa Cruz Biotechnology). Specific immunolabeling was obtained by using horseradish peroxidase-conjugated secondary antibodies, followed by the SuperSignalWest Pico chemiluminescence detection system (Pierce, Rockford, IL, USA).

### Immunohistochemistry

Mice were deeply anesthetized with sodium pentobarbital (100 mg/kg, i.p.) and transcardially perfused with 0.9% NaCl in 0.1 m PBS, pH 7.4 followed by 4% PFA in 0.1 m PBS (pH 7.4). The brains were then dissected and postfixed in 4% PFA at 4 °C for 48 h. Brain tissues were cryoprotected, embedded in OCT and frozen. Then 40-*μ*m-thick transverse cryosections were collected. Following primary antibodies were used: rabbit polyclonal, and mouse monoclonal anti-GFP antibodies (1 : 1000 dilution; Abcam, Cambridge, MA, USA) to identify EYFP, anti MBP antibody (1 : 500; Sternberger and Sternberger), and rabbit polyclonal anti-human Olig2 antibody (1 : 500; Abcam).

For the peroxidase immunohistochemistry, tissue sections were blocked with 10% normal goat serum and then incubated in 0.1 m PBS containing 0.1% Triton-X and the primary antibody for 16–18 h at 4 °C. Sections were then incubated in HRP-conjugated goat anti-rabbit or HRP-conjugated goat anti-mouse secondary antibodies (1 : 200; Jackson ImmunoResearch Laboratories, West Grove, PA, USA) for 2 h at room temperature. Diaminobenzidine (0.5 mg/ml) was used to visualize the reaction. Finally, sections were dehydrated and cover-slipped with Entellan mounting medium (BDH Chemicals, Toronto, ON, Canada).

Brain sections for fluorescent immunohistochemistry were blocked with 10% normal goat serum (Jackson ImmunoResearch Laboratories) and then incubated in PBS containing 0.1% Triton-X and the primary antibody overnight. Sections were then incubated for 2 h at room temperature in a mixture of Alexa 546-conjugated goat anti-rabbit IgG, Alexa 488-conjugated goat anti-mouse IgG and Alexa 643-conjugated goat anti-guinea pig IgG (Molecular Probes Inc., Eugene, OR, USA), at 1 : 2000 dilution. Sections were cover slipped in non-fluorescing mounting medium (Fluorsave Reagent, Calbiochem, La Jolla, CA, USA).

### Data analysis

Photomicrographs of p38*α* fl/fl and p38*α* CKO brain sections were captured under identical setting, with a SPOT Cooled Color digital camera (Diagnostic Instruments Inc., Mawah, NJ, USA), mounted on a Zeiss microscope (Pleasanton, CA, USA) and assembled in Adobe Photoshop (version 9). For quantification of MBP staining intensity, ImageJ (Bethesda, MD, USA) was used. Selected areas (1 mm × 1 mm or 2 mm × 2 mm) were used to analyse statistical significances.

Electron microscopic photomicrographs were analyzed for myelination by calculation of the G (*g*) ratio (the ratio of axon circumference to myelin circumference). Briefly, high magnification images of myelinated axons were obtained and imported to the ImageJ software for measuring the diameter of the axons. The *g*-ratio was calculated as the diameter of the axon (*a*) divided by diameter of the myelinated axon caliber (*A*): *g*-ratio=*a*/*A*. Thus, the smaller the *g*-ratio is, the thicker is the myelin sheath layer. All of the data are represented as mean±S.E.M. Each experimental group had at least eight mice. Statistical assessments were analyzed using ANOVA with *post-hoc* Tukey's test when multiple group comparisons were made and Student's *t*-test when two independent groups were compared. *P*-values of <0.05 were considered significant.

## Abbreviations

CC: corpus callosum
CKO: conditional knockout
CNS: central nervous system
EGFP: enhanced green fluorescent protein
GM: growth medium
MAPK: mitogen-activated protein kinase
MBP: myelin basic protein
MS: multiple sclerosis
NG2: nerve/glial antigen 2
OF: observational fields
OL: oligodendrocyte
OPC: OL progenitor cells
P: postnatal day
PLP: proteolipid peptide
p38*α* fl/fl: p38*α*-floxed
St: striatum
